# Age-Dependent Changes in Taurine, Serine, and Methionine Release in the Frontal Cortex of Awake Freely-Moving Rats: A Microdialysis Study

**DOI:** 10.3390/life15020295

**Published:** 2025-02-13

**Authors:** Cristina Cueto-Ureña, María Jesús Ramírez-Expósito, María Pilar Carrera-González, José Manuel Martínez-Martos

**Affiliations:** Experimental and Clinical Physiopathology Research Group CTS-1039, Department of Health Sciences, School of Health Sciences, University of Jaén, E-23071 Jaén, Spain; ccueto@ujaen.es (C.C.-U.); mramirez@ujaen.es (M.J.R.-E.); pcarrera@ujaen.es (M.P.C.-G.)

**Keywords:** taurine, frontal cortex, methionine, serine, microdialysis

## Abstract

Brain function declines because of aging and several metabolites change their concentration. However, this decrease may be a consequence or a driver of aging. It has been described that taurine levels decrease with age and that taurine supplementation increases health span in mice and monkeys, finding taurine as a driver of aging. The frontal cortex is one of the most key areas studied to know the normal processes of cerebral aging, due to its relevant role in cognitive processes, emotion, and motivation. In the present work, we analyzed by intracerebral microdialysis in vivo in the prefrontal cortex of young (3 months) and old (24 months) awake rats, the basal- and K^+^-evoked release of taurine, and its precursors methionine and serine. The taurine/serine/methionine (TSM) ratio was also calculated as an index of transmethylation reactions. No changes were found in the basal levels of taurine, serine, or methionine between young and aged animals. On the contrary, a significant decrease in the K^+^-evoked release of serine and taurine appeared in aged rats when compared with young animals. No changes were seen in methionine. TSM ratio also decreased with age in both basal- and K^+^-stimulated conditions. Therefore, taurine and its related precursor serine decrease with age in the frontal cortex of aged animals under K^+^-stimulated but not basal conditions, which supports the importance of the decline of evoked taurine in its functions at the brain level, also supporting the idea proposed by other authors of a pharmacological and/or nutritional intervention to its restoration. A deficit of precursors for transmethylation reactions in the brain with age is also considered.

## 1. Introduction

Aging is a very complex physiological process that affects all organs, usually by decreasing their function, because of genomic instability, mitochondrial dysfunction, decrease in stem cell reserves, accumulation of senescent cells, or alteration of endocrine axes, among others [1,2,3,4]. The age-associated decrease in the function of the different organs is therefore reflected in multiple changes in the concentration of endogenous metabolites, hormones, and micronutrients [5,6,7]. The aged brain is characterized by several neurochemical modifications involving structural proteins, neurotransmitters, neuropeptides, and related receptors. Alterations in the neurochemical indices of synaptic function are indicators of the age-related impairment of central functions, such as locomotion, memory, and sensory performance [8]. These changes may be a consequence or a driver of aging [9].

Taurine or 2-aminoethanesulfonic acid is a non-proteinogenic amino sulfonic acid that is widely distributed in animal tissues [10,11,12], but that plays important multiple roles in the central nervous system [13]. Thus, it acts as a neurotransmitter, as a trophic factor in CNS development, and as a regulator of calcium transport and homeostasis [14,15,16,17,18,19].

As a neurotransmitter, taurine meets the criteria to be considered as such, with evidence of its presence in neurons, release after stimulation, physiological response, specific receptors, and inactivation mechanisms [14]. The presence of metabotropic taurine receptors that are negatively coupled to the phospholipase C signaling pathway via inhibitory G proteins is also proposed [15,20]. As a trophic factor in CNS development, it is well known that kittens from taurine-depleted mothers exhibit a delay in the migration of cells in the cerebellum and in the visual cortex [21,22]. As a regulator of calcium, taurine also regulates intracellular calcium homeostasis by inhibiting calcium entry and release from internal stores [23,24].

Taurine is a compound that also acts as an osmolyte [22,25,26], neuromodulator [22], and neuroprotectant [8,27,28]. In this way, taurine as a neuroprotective agent can prevent glutamate-induced neuronal injury and protect against H_2_O_2_-induced cell injury. This neuroprotective function is due to its role in reducing the intracellular concentration of free calcium and its antioxidant ability. In this regard, taurine can also shift the ratio of the anti-apoptotic protein Bcl-2 and the pro-apoptotic protein Bax towards cell survival by inhibiting glutamate-induced calcium activation and subsequent the heterodimerization of Bcl-2 and Bax proteins leading to the apoptosis cascade [8].

**Figure 1:**
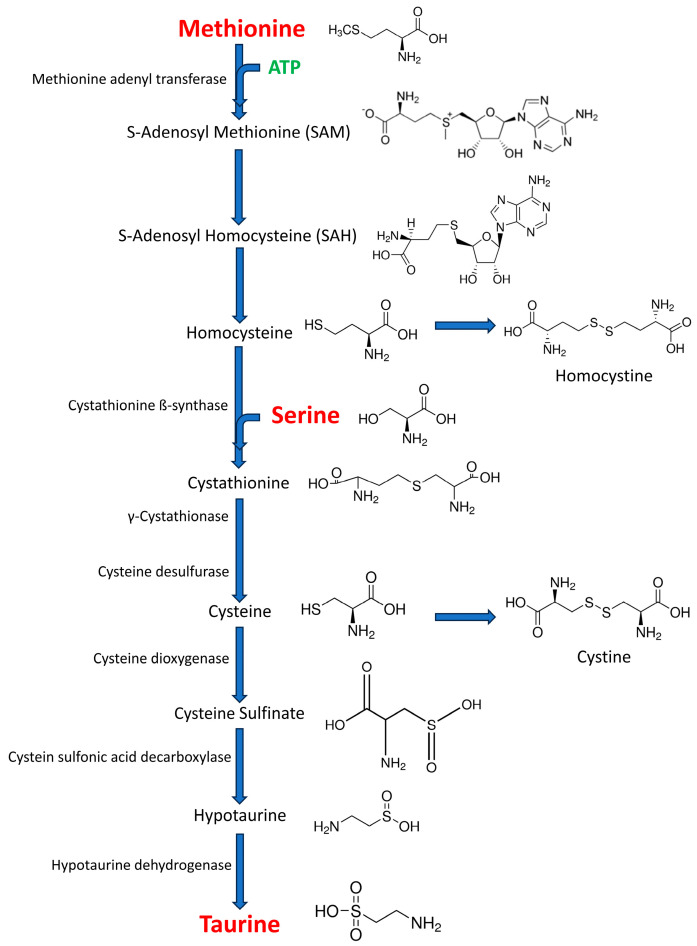
Taurine biosynthesis by cysteine sulfonic acid decarboxylase route, the major route of taurine biosynthesis in the central nervous system. This route begins with methionine and/or cysteine amino acids. Methionine is transformed into cysteine through four intermediate steps, involving the formation of S-adenosyl methionine (SAM), S-adenosylhomocysteine (SAH), homocysteine, and cystathionine. Cystathionine results from the condensation of one homocysteine molecule with one serine molecule, and this reaction is catalyzed by a cystathionine ß-synthase. Cystathionine gives rise to one cysteine molecule following the action of two enzymes in succession: γ-cystathionase and cysteine desulfurase. The cysteine generated by this pathway or obtained through the diet can be used as a substrate for taurine generation. In the major pathway, cysteine is converted into cysteine sulfonic acid by cysteine dioxygenase. The product of this reaction is then converted into hypotaurine by cysteine sulfonic decarboxylase, and hypotaurine in taurine by hypotaurine dehydrogenase (modified from [30]).

Taurine can be synthesized in the body (Figure 1) from cysteine and methionine through a series of enzymatic reactions [29]. Methionine is first converted to S-adenosylmethionine (SAM), which is then converted to S-adenosylhomocysteine (SAH) and then to homocysteine. Homocysteine can then be converted to cystathionine by the enzyme cystathionine β-synthase using serine as a substrate. Cystathionine is then converted to cysteine by the enzyme cystathionase. Cystathionine results from the condensation of one homocysteine molecule with one L-serine molecule, and this reaction is catalyzed by a cystathionine ß-synthase. Thus, serine levels can, theoretically, regulate taurine biosynthesis [30]. Cysteine can then be converted to hypotaurine and then to taurine.

Taurine deficiency has been linked to several neurological disorders, including epilepsy [20,31], Alzheimer’s disease [32,33], and Parkinson’s disease [34,35,36,37]. Taurine supplementation has been shown to improve cognitive function and reduce oxidative stress in animal models of these diseases [38,39]. It has also been shown that taurine may have therapeutic applications in other neurological disorders, such as stroke, traumatic brain injury, and spinal cord injury [7,37,40,41]. Taurine supplementation has been shown to reduce neuronal damage and improve functional recovery in animal models of these conditions.

In the present report, we analyzed basal- and K^+^-evoked levels of taurine in the frontal cortex of awake, freely moving rats, by intracerebral microdialysis in vivo in young and aged animals, as well as the basal and evoked levels of the precursor amino acids methionine and serine, together with the TSM transmethylation index. The frontal cortex is one of the major areas studied to know the normal processes of cerebral aging due to its relevant role in cognitive processes, emotion, and motivation [42]. Here, we support a decline in taurine brain functions with age, which could be responsible, at least in part, for the neurological disorders that occur during aging. A deficit of precursors for transmethylation reactions in the brain with age is also considered.

## 2. Materials and Methods

### 2.1. Animals

This study employed thirty 3-month-old (235 ± 12 g body weight) and thirty 24-month-old (524 ± 18 g body weight) male Wistar rats. The animals were provided by the University of Jaén’s animal care facility and housed in a controlled setting with a consistent temperature (25 °C) and 12 h light/12 h dark cycles. All the animals had unlimited access to food and water. The use and care of animals in the experimental procedures were carried out in compliance with the European Community Council Directive (2010/63/EU).

### 2.2. Microdialysis Procedure

Microdialysis in the frontal cortex was performed with an I-shaped probe prepared from polyacrylonitrile/sodium methalyl sulfonate copolymer as previously described [43]. The characteristics of the probe were i.d., 0.22 mm, o.d. 0.31 mm, and the exposed tip of the dialysis membrane was 4 mm long. The in vitro recovery of the membrane for methionine, serine, and taurine (n = 10) was 9.85 ± 2.75, 17.87 ± 2.19 and 19.45 ± 1.95%, respectively.

**Figure 2:**
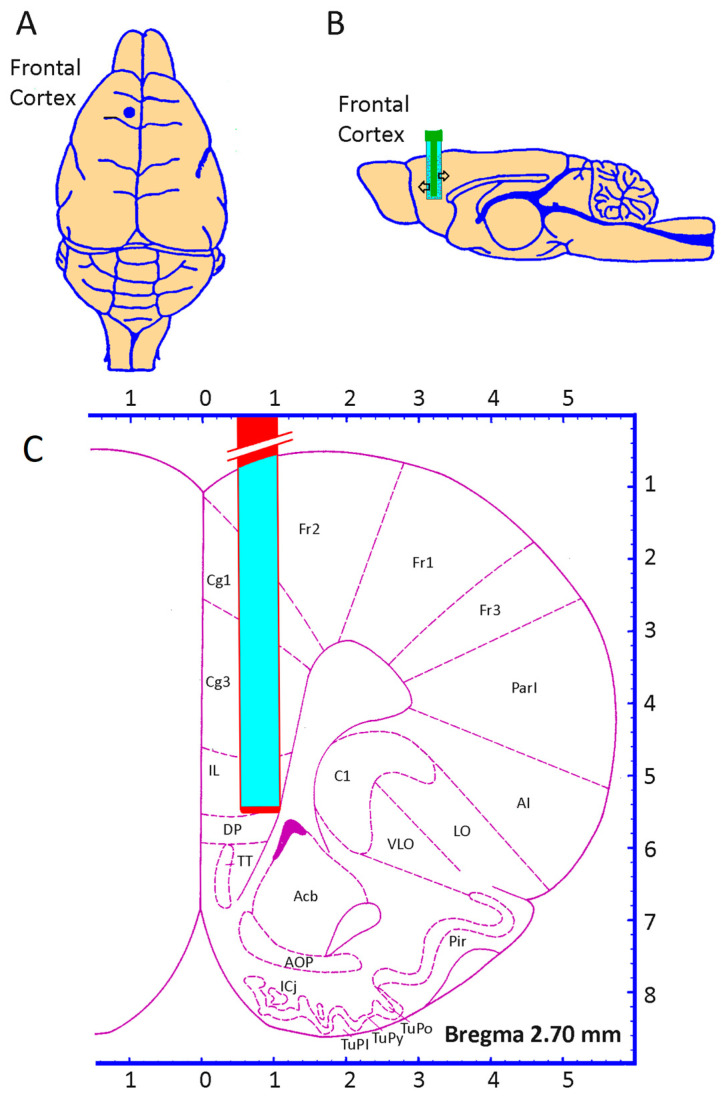
Dorsal (**A**) and sagittal (**B**) representation showing the localization of the probe in the frontal cortex. (**C**) Schematic diagram of a coronal section of the brain showing the localization of the microdialysis probe in the frontal cortex of the rat according to stereotaxic coordinates.

Under intraperitoneal equithesin anesthesia (2 mL/kg), the animals were placed in a stereotaxic instrument. A hole was drilled through the skull bone and the dura was exposed. The microdialysis probe was then slowly introduced into the frontal cortex. Stereotaxic coordinates for the microdialysis tip from bregma were anterior, 2.7 mm; lateral, 0.8 mm; and dorsal −5.0 mm from the dura (Figure 2). The microdialysis probe was secured with dental cement. Three tiny stainless steel screws were attached to the skull bone to give the dental cement a better grip. After surgery, the animals were housed under a 12/12 h night–day cycle in separate cages at room temperature (20–25 °C) with water and standard food pellets freely available. The perfusion experiments were performed 18–24 h after the implantation of the probes.

An artificial cerebrospinal fluid (CSF) containing Cl^−^ 134.7 mM, Na^+^ 127.7 mM, K^+^ 2.6 mM, Ca^2+^ 1.3 mM, and Mg^2+^ 0.9 mM at a flow rate of 2 μL/min was perfused. Perfusates collected during the first 60 min were discarded and the next 30 min samples were collected and immediately frozen at −80 °C until the analysis and considered basal levels. The effects on K^+^-evoked release were evaluated by adding K^+^ 100 mM at the next 30 min. The increase in K^+^ 100 mM in the perfusion fluid was compensated by a proportional reduction in Na^+^ to maintain osmolarity [43]. Animals exhibited normal behavior in the freely moving rat during the perfusion procedures. Each animal was used once.

Routinely, after the completion of the experiments, all the brains were studied histologically exclusively to verify the anatomical localization of the microdialysis probes and the precise perfusion sites. In any case, those experiments that presented a poor localization of the probes or any type of lesion in the perfused areas, whether inflammatory or mechanical, were excluded [44].

### 2.3. Amino Acids Determination

The methionine, serine, and taurine content were assayed by HPLC coupled to a fluorescence detection system, as previously described [45,46]. Data were processed with the TotalChrom WorkStation ver. 6.3.1 software from Perkin-Elmer (Madrid, Spain). Concentrations were expressed as pmoles of amino acid per microliter of perfusate. The TSM ratio was calculated as the ratio between a hundred times the taurine level and the product of the levels of serine and methionine. It is an indicator of the amino acids involved in transmethylation processes, which are altered in diseases such as Alzheimer’s disease [47] and psychoses [48].

### 2.4. Statistical Analysis

All the values represent the mean ± standard error of the mean (SEM). The data were analyzed by two-way analysis of variance and Student–Newman–Keuls post hoc test, using the Statgraphics Centurion 19 software. Values of *p* < 0.05 were considered significant.

## 3. Results

### 3.1. Basal Release of Methionine, Serine, and Taurine

**Figure 3:**
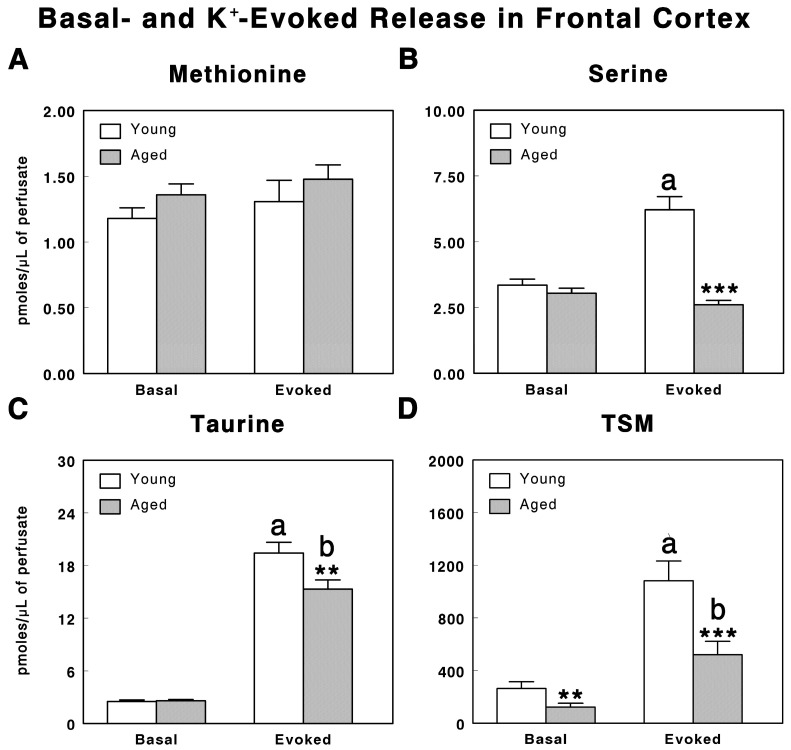
Basal- and potassium-evoked release of methionine (**A**), serine, (**B**) and taurine (**C**), and taurine/serine/methionine (TSM) ratio (**D**) in the frontal cortex of the young (3 months old) and aged (24 months old) rats obtained by intracerebral microdialysis in vivo in awake, freely moving animals. The values are expressed in pmoles per microliter of perfusate (mean ± SEM; n = 30; ^a^ *p* < 0.001 basal vs. evoked values in the young animals; ^b^ *p* < 0.001 basal vs. evoked values in the aged animals; ** *p* < 0.01, basal values in the young vs. aged animals and *** *p* < 0.001 evoked values in the young vs. aged animals).

Figure 3A–C show the basal levels of methionine, serine, and taurine in the frontal cortex of the awake young and aged animals. In the young animals, the basal methionine levels were 1.179 ± 0.08 pmoles/µL of perfusate, whereas in the aged animals, the values were 1.360 ± 0.08 pmoles/µL of perfusate (Figure 3A), without significant differences. Similarly, serine levels in the young animals were 3.349 ± 0.23 pmoles/µL of perfusate, and in the aged animals, they were 3.043 ± 0.19 pmoles/µL of perfusate (Figure 3B). Regarding taurine, in the young animals, we found 2.512 ± 0.18 pmoles/µL of perfusate, whereas in the aged animals, 2.585 ± 0.14 pmoles/µL of perfusate were found (Figure 3C). No significant differences were also found in the serine and taurine levels with age.

### 3.2. Methionine, Serine, and Taurine Release Evoked by 100 mM K^+^

In the young animals, K^+^ 100 mM added to the artificial CSF produced a significantly increased evoked release of serine (F(1, 116) = 14.46, *p* < 0.001; n = 30; Figure 3B) and taurine (F(1, 116) = 409.5, *p* < 0.001; n = 30; Figure 3C), but not of methionine (Figure 3A). Thus, serine reaches values of 6.212 ± 0.50 pmoles/µL of perfusate after potassium stimulation and taurine reaches values of 19.41 ± 1.23 pmoles/µL of perfusate after K^+^ stimulation. After K^+^ 100 mM, methionine reaches values of 1.307 ± 0.16 pmoles/µL of perfusate, without significant differences between basal and evoked values.

### 3.3. TSM Index

The taurine/serine/methionine ratio, an index of transmethylation processes, was significantly decreased (F(1, 116) = 6.363, *p* < 0.01; n = 30) in the aged animals in comparison with young animals when calculated in both the basal- and K^+^-evoked conditions (Figure 3D). Thus, the young animals showed basal TSM values of 263.7 ± 52.79, whereas the aged animals showed basal TSM values of 122.91 ± 29.24. Similarly, the TSM index under K^+^-evoked conditions showed values of 1081.96 ± 150.08 in the young animals, whereas in the aged ones, values of 519.25 ± 101.64 were found (F(1, 116) = 8.114, *p* < 0.001; n = 30).

## 4. Discussion

The important role that taurine may have in the normal performance of nervous system functions, and how its deficit, as a consequence of aging, may be responsible in some way for the multiple nervous dysfunctions associated with aging has recently been highlighted [9,49,50]. Here, we have evaluated, in awake and freely moving animals, how the basal and evoked levels of taurine and other related amino acids are modified as a consequence of aging to corroborate these findings and support the idea of the therapeutic possibilities of taurine administration to ameliorate these deficiencies. In fact, and according to our results, serine administration could also be useful, alone or in combination with taurine, as an additional therapeutic approach [51].

Regarding taurine, a very significant decline with age in the blood concentrations of the amino acid taurine has recently been described in several species, including mice, monkeys, and humans [9]. In fact, in humans, it has been shown that the decrease in serum taurine levels can be as much as 80% in an aging person compared to a young individual [9]. In the mouse, the results found show that taurine deficiency is a driver of aging because if reversed, it increases the lifespan of these animals [9]. Also, in animals with a congenital taurine deficiency, accelerated aging, decreased bone density, much poorer neuromuscular coordination, compromised muscle strength, a significant increase in anxiety, and a very significant decrease in memory were demonstrated [9]. Sex differences at the brain level have even been suggested in relation to behavior [32,52,53,54]. Thus, it has been shown that in aged female mice, which show increased levels of anxiety but decreased exploratory ability, taurine administration decreased depression-like behaviors compared to non-taurine-treated controls [9].

In humans, attempts have also been made to determine the influence of the blood levels of taurine and its metabolites on various health-related variables, and associations have been found at several levels, including lower body mass index; lower waist–hip ratio, and thus lower obesity; lower prevalence of diabetes; and lower blood glucose in people with elevated taurine levels [55]. Although these associations do not imply causality, they do show that taurine deficiency contributes to aging, as the amount of taurine in the blood and tissues decreases with age, as it does in other species [9].

In this work, we show that an analysis of the frontal cortex of the aged rat shows normal basal levels of both taurine and its amino acid precursors serine and methionine. However, the release evoked with 100 mM K^+^ shows a remarkable decrease in taurine levels in the aged animals, even more clearly the decrease in the release of its precursor serine, whose release in the young animals was particularly important. In fact, the evoked serine release was completely abolished in the aged animals. Given that serine is a precursor that can regulate taurine biosynthesis, it is possible that a deficit in this amino acid may be responsible, to some extent, for the decrease in the taurine levels found here, mainly considering that the precursor methionine did not show any alteration either in its basal or evoked levels because of aging. Similarly, the TSM index, a marker of the transmethylation processes occurring during metabolism, is found to be decreased with aging, suggesting a deficit of methyl groups for the various metabolic transmethylation reactions in aged animals.

As previously stated, taurine is synthesized in cells from the sulfur-containing amino acid methionine in a metabolic pathway involving a series of sulfur-containing molecules in which demethylation, decarboxylation, and oxidation reactions occur. These reactions occur in most tissues and could be altered with aging [56]. Furthermore, in the CNS, in the transformation of cysteine to taurine there are really two pathways involved. In one of them, cysteine is converted to cysteinosulfinic acid, which is subsequently converted to cysteic acid and finally transformed to taurine by a cysteic acid-dependent decarboxylase [57]. In the other metabolic pathway, cysteinosulfinic acid is converted to hypotaurine and finally to taurine, in a pathway that has been little studied in terms of its regulatory mechanism and the enzymes involved in the transformations [21]. In the trajectory of taurine biosynthesis, the enzyme mainly studied is cysteinosulfinic decarboxylase, which transforms cysteinosulfinic acid into hypotaurine, this being the limiting stage in some tissues [58]. This transformation is of importance in the liver and brain during development, where despite a deficit of the enzyme, there is a high concentration of taurine [57,59]. Thus, the concentration of taurine is also determined by the dietary intake, the need for taurine in the diet, in principle, being much higher in young animals than in old ones [60]. Further studies obtaining information on the possible modifications of the enzymatic reactions involved in the biosynthesis of taurine at the intracellular level or ex vivo immunohistochemistry could shed light on the physiopathology of aging effects.

However, it must also be considered that taurine is ubiquitous in nature and its distribution and amount differ in different biological organisms [14]. Its antioxidant and osmoregulatory roles have been widely known [61,62]. Its synthesis may differ in distinct species, which is important in its physiological role and in pathological conditions. Taurine is an essential nutrient in felines, such as cats, where its absence in the diet causes several abnormalities [63,64]. In humans, taurine can be considered as a conditionally essential nutrient, especially in children receiving total parenteral nutrition for prolonged periods of time [60]. In premature infants who are formula-fed, its supplementation is recommended. In adults, its clinical importance is yet to be demonstrated in different pathologies [50]. Thus, the decreased bioavailability of taurine under stimulated conditions in nervous tissue with age found here could explain the unbalance in the physiological processes in which taurine is involved and also why taurine administration could revert these malfunctions.

Animal studies have demonstrated that the chronic supplementation of taurine in aged mice decreased the age-dependent decline in spatial memory acquisition and retention [65,66,67,68]. Concomitant with the amelioration in cognitive function, taurine caused significant alterations in the GABAergic and somatostatinergic systems. These changes included increased levels of the neurotransmitters GABA and glutamate, increased expression of both isoforms of glutamate decarboxylase (GAD 65 and 67) and the neuropeptide somatostatin, decreased hippocampal expression of the β3 subunits of the GABA_A_ receptor, increased expression in the number of somatostatin-positive neurons, increased amplitude and duration of population spikes recorded from CA1 in response to Schaefer collateral stimulation, and enhanced paired-pulse facilitation in the hippocampus [8]

Taurine has been proposed as a potential therapeutic tool to treat the neurodegenerative disorders of Alzheimer’s and Parkinson’s diseases [8]. Alzheimer’s disease is characterized by the accumulation of β-amyloid peptides in neurons and the accumulation of extracellular glutamate leading to excitotoxicity and cell death [69]. The administration of taurine in rats activates GABA_A_ receptors [70] and protects cortical and hippocampal neurons from the accumulation and toxicity of β-amyloid peptide and extracellular glutamate accumulation [71]. In patients with Parkinson’s and Alzheimer’s disease [72], the concentration of taurine in the cerebrospinal fluid is lower than in control subjects [36], so taurine supplementation was studied to help in the treatment of these diseases. However, other research shows an interaction between taurine and L- Dopa in the case of Parkinson’s disease and with Tau protein in the case of Alzheimer’s disease, recommending further studies before its use in patients with these diseases [33,73]. However, taurine derivatives such as tauroursodeoxycholic acid (TUDCA) or homotaurine have been studied for the treatment of Parkinson’s disease and Alzheimer’s disease, since TUDCA increases survival in the cell transplants of substantia nigra in the case of Parkinson’s and both substances decrease the aggregation of β-amyloid protein in the case of Alzheimer’s [35,74,75]. Niemann–Pick disease type C1, an inherited fatal disorder, is characterized by a defect in cholesterol transport and progressive neurodegeneration. In this model, taurine can rescue neurons from apoptosis by inhibiting caspase 9 activation [76]. In the brains of humans and animals with epilepsy, taurine concentrations have been shown to be decreased [77]. These deficits are caused by prolonged damage caused by seizures and by the persistence of a state of hyperexcitability in the brain. Taurine analogs such as homotaurine, taltrimide, acamprosate, and tauromuthine function as anticonvulsants and are used in the treatment of human epilepsy [31]. Interestingly, taurine plays a role in the cognitive deficit caused by lead poisoning. The accumulation of this metal leads to a decrease in neuronal activity, a decrease in the number of dendritic spines [78], and a blockade of the NMDA receptor [79]. Nutritional supplementation with taurine during pregnancy protects against these known damages due to lead accumulation during fetal development [80]. More recently, it has been described that taurine treatment may upregulate TREM2 to protect against microglia over-activation by decreasing the accumulation of phospho-tau and Aß, providing insight into a novel preventive strategy in neurodegeneration [81].

Although here we have focused primarily on the role of serine as a precursor of taurine, serine also has an important role as a precursor of glycine, as well as a neurotrophic factor [82,83]. Thus, serine has been shown to be required for cell proliferation, for neuronal development, and for various brain functions [82,84]. Serine also regulates the release of several brain cytokines in neuropathological conditions and enables the recovery of cognitive function, improves cerebral blood flow, inhibits inflammation [85], and promotes remyelination [37,41,86] in addition to other neuroprotective effects [83,87,88,89]. Serine has also been used to treat epilepsy, schizophrenia, psychosis, Alzheimer’s disease, and other neurological diseases [83,87,88,89]. Using experimental animals, serine interventions have demonstrated significant therapeutic effects, and the substance is also very safe to administer [90]. The decrease in serine bioavailability under stimulated conditions in the aged animals shown here could also condition its physiological functions.

It should also be noted that a small part of L-serine is metabolized in the human body into D-serine by the enzyme serine racemase [91]. The physiological roles of D-serine in the development of the central nervous system and in pathological conditions related to neuropsychiatric and neurodegenerative disorders related to NMDA receptor dysfunction are well known [92,93]. Decreased L-serine levels may, therefore, also alter D-serine levels. Indeed, D-serine levels and the D-serine/L-serine ratio have been shown to decrease in the brains of patients with schizophrenia, pediatric encephalopathy, and Alzheimer’s disease [90,94,95]. In experimental animals, the reduced synthesis of both D-serine and L-serine has been shown in a mouse model of Alzheimer’s disease and in relation to alterations in synaptic plasticity and memory [90].

As a limitation, we should not lose sight of the fact that there are multiple intracellular mechanisms involved in the aging processes that can, in some way, modify the amino acid levels found in our study. For example, the increased reuptake of metabolites from the synaptic cleft may promote a lower availability of these metabolites for analysis by microdialysis so that the deficiencies detected here would not be compensated by taurine supplementation [96,97].

Finally, our results also show a decrease in the TSM ratio, suggesting a decrease in the transmethylation processes during aging. During transmethylation processes, methionine is adenylated to SAM in a reaction catalyzed by S-adenosylmethionine synthase and SAM is a methyl donor for biomolecules such as DNA, RNA, proteins, and monoamine neurotransmitters [40]. The demethylation of SAM by the methyl transferase generates SAH, which is finally hydrolyzed by SAH hydrolase to form homocysteine and adenosine. Homocysteine can be remethylated back to methionine through remethylation processes [34]. Transmethylation and remethylation are the two pathways closely interconnected with the transsulfuration pathway [98], a process by which homocysteine produced by transmethylation is irreversibly converted to cysteine. Taken together, failures at the level of these complex metabolic pathways because of aging may also be behind the neurological alteration that characterizes advanced age.

Many studies have, therefore, shown a significant role for taurine in the homeostasis of the nervous system and other organs [13]. Our results confirm, using in vivo brain microdialysis in awake animals, that the evoked release of taurine (and its precursor serine) is strongly decreased because of aging, although the basal release is not modified. The decrease in the evoked levels of these amino acids may be responsible to some extent for the age-related alterations in nerve function. Therefore, the proposed intervention of nutritional or pharmacological taurine supplementation, which has been shown to be effective in mice and monkeys [9], could also be effective in humans, although it needs to be carefully studied and related to a putative additional/alternate serine intervention. Due to methionine, taurine, serine, and other intermediaries participating in several metabolic routes involving transmethylation, remethylation, and transsulfuration vias, the complexity of nutritional/pharmacological interventions related to the administration of these amino acids must also be considered.

## 5. Conclusions

Aging decreases the function of most organs in individuals, including the nervous system. This may be reflected in the modified levels of different metabolites, which may be a consequence or driver of aging. Among these drivers, taurine, an amino acid with multiple brain functions and whose deficiency has been linked to multiple neurological disorders, has recently been proposed. This study offers valuable insights into the age-related changes in neurotransmitter metabolism, focusing primarily on taurine, serine, and methionine in the frontal cortex of freely moving rats. The findings indicate that while the basal levels of these metabolites remain unchanged, their release in response to potassium (K^+^) stimulation declines significantly with age, particularly for taurine and serine. Additionally, the observed decrease in the taurine/serine/methionine (TSM) ratio supports the hypothesis of impaired transmethylation reactions due to aging. These results suggest a potential role for taurine in age-related cognitive decline and underline the need for pharmacological or nutritional strategies to restore its function. However, further research is necessary to determine whether the decrease in taurine is a cause or a consequence of age-related brain dysfunction. Investigating whether taurine supplementation can effectively counteract these deficits in vivo and improve cognitive outcomes in aging models is crucial. Furthermore, exploring the underlying mechanisms behind the reduced evoked release could provide deeper insights into the neurochemical changes associated with aging.

## Data Availability

The raw data supporting the conclusions of this article will be made available by the authors on request.
